# Editorial: Neurological comorbidity in metabolic syndrome

**DOI:** 10.3389/fnins.2023.1263570

**Published:** 2023-08-15

**Authors:** Matilde Otero-Losada, Anna Marseglia, Eduardo Blanco Calvo, Francisco Capani

**Affiliations:** ^1^Centro de Altos Estudios en Ciencias Humanas y de la Salud, Universidad Abierta Interamericana, Consejo Nacional de Investigaciones Científicas y Técnicas, CAECIHS.UAI-CONICET, Buenos Aires, Argentina; ^2^Department of Neurobiology, Care Sciences and Society, Division of Clinical Geriatrics, Center for Alzheimer Research, Karolinska Institutet, Stockholm, Sweden; ^3^Departamento de Psicobiología y Metodología de las Ciencias del Comportamiento, Instituto de Investigación Biomédica de Málaga (IBIMA), Universidad de Málaga, Málaga, Spain; ^4^Facultad de Ciencias de la Salud, Instituto de Ciencias Biomédicas, Universidad Autónoma de Chile, Santiago de Chile, Chile

**Keywords:** metabolic syndrome type II diabetes, neurological comorbidity, insulin resistance, aging, oxidative stress, inflammation, enriched environment (EE), chronic cerebral hypoperfusion

Metabolic syndrome (MetS) is a cluster of cardiovascular and metabolic conditions. It is prevalent in older adults and represents a substantial public health outlay worldwide (Otero-Losada et al., 2016). Its typical features—atherogenic dyslipidemia, oxidative stress, and inflammation—lead to microvascular dysfunction and chronic cerebral hypoperfusion (Herrera et al., 2018), and neurodegenerative and cognitive impairments (Maiuolo et al., 2021).

Insulin resistance is a central factor in MetS and, likely, a key link between MetS and Alzheimer's disease (AD) (Kim and Feldman, 2015; Etchegoyen et al., 2018). It is associated with cognitive and non-cognitive abnormalities, impaired visuospatial skills, attention deficit, and psychomotor disturbances (Ma et al., 2015). There are two major insulin signal transduction pathways. The insulin-phosphatidylinositol-4,5-bisphosphate 3-kinase (PI3K)/AKT pathway, which manages the metabolic effects, and the Ras/MAPK pathway, which regulates cell growth, survival, and gene expression (Zhang et al., 2019). Insulin signal transduction failures impair neuroplasticity and favors premature aging, neuroinflammation, astrocytosis and demyelination, leukoaraiosis, loss of synapses with dystrophic neurites formation, and microvascular disease (Ashpole et al., 2015; Sedzikowska and Szablewski, 2021). Insulin resistance promotes neuroinflammation, amyloid β-peptide deposition, and aberrant tau phosphorylation (Wei et al., 2021). The other way around, amyloid β aggregates—pathognomonic of AD—have been observed in diabetes and may favor diabetes pathology (Stanciu et al., 2020). AD has indeed been regarded as a degenerative metabolic disease caused by brain insulin resistance, overlapping with molecular, biochemical, pathophysiological, and metabolic dysfunctions occurring in MetS, type 2 diabetes (T2D), and non-alcoholic fatty liver disease (de la Monte, 2017). Neurodegenerative dementia shows synaptic and neuronal loss, microgliosis, and defective glucose metabolism and insulin-PI3K/Akt signaling in the brain (Merluzzi et al., 2018).

Neurological diseases reduce both life quality and expectancy and improving our knowledge of MetS implications at the cerebral level is needed. Identifying, designing, or developing therapeutic strategies to improve the metabolic-neurological connection will ultimately reduce the neurological impact of MetS.

This Research Topic addresses issues like changes in the hippocampus in T2D; how MetS affects cognition in midlife; probiotic supplementation benefits in experimental dementia; hypercholesterolemia and Alzheimer's disease; protein misfolding in MetS; and how environmental enrichment favors neurodevelopment and plasticity in metabolic syndrome throughout life.

The following subjects are covered:

- *The Metabolic Syndrome Is Associated with Lower Cognitive Performance and Reduced White Matter Integrity in Midlife: The CARDIA Study* (Dintica et al.): The authors examined the relationship between MetS and cognitive deficits in midlife (mean age 50) over a 5-year follow-up, within the prospective Coronary Artery Risk Development in Young Adults (CARDIA) study. A cognitive battery was administered and a subgroup of participants underwent brain MRI. MetS was associated with lower cognition and microstructural brain alterations already at midlife. MetS should be targeted earlier in life in order to prevent adverse brain and cognitive outcomes (Whitmer et al., 2005; Yaffe et al., 2020).
- *Connecting the Dots Between Hypercholesterolemia and Alzheimer's Disease: A Potential Mechanism Based on 27-Hydroxycholesterol* (Wu et al.): Hypercholesterolemia is a risk factor for the most common cause of dementia, Alzheimer's disease (AD), though cholesterol-lowering drugs show mixed results in improving cognitive function. Yet, drugs that target cholesterol exocytosis and conversion improve AD pathology. This chapter examines cholesterol metabolism in the brain, focusing on oxysterols' role. 27-hydroxycholesterol (27-OHC) is the major peripheral oxysterol that flows into the brain and not only affects amyloid-β production and elimination but other pathogenic mechanisms of AD as well. The mechanisms involved in the 27-OHC role in AD pathology are still to be unraveled in order to identify new therapeutic strategies for AD (González et al., 2020; Ding et al., 2021).
- *The Impact of Probiotic Supplementation on Cognitive, Pathological and Metabolic Markers in a Transgenic Mouse Model of Alzheimer's Disease* (Webberley et al.): Supplementation with probiotic bacteria is emerging as a preventive strategy for neurodegeneration and metabolic syndrome. The authors show the impact of the Lab4b probiotic consortium on (i) cognitive and pathological markers of AD progression and (ii) metabolic status in 3xTg-AD mice subjected to a metabolic high-fat diet challenge. They show that a 12-week probiotic supplementation improved performance in the novel object recognition test, increased hippocampal neuronal spine density, and improved brain and systemic anti-inflammatory responses. The data presented support the neuroprotective potential of probiotic supplementation (Fan and Pedersen, 2020; Zhu et al., 2021).
- *Changes in the structure, perfusion, and function of the hippocampus in type 2 diabetes mellitus* (Li et al.): This chapter shows the use of multimodal MRI methods to explore the structure, perfusion, and function of the bilateral hippocampus in T2D. The volume and perfusion of the hippocampus are decreased in T2D patients related to chronic hyperglycemia. Local spontaneous neural activity and coordination are increased in the hippocampus of T2D patients, possibly as an adaptive compensation for cognitive decline. The study provides reliable neuroimaging evidence for the diagnosis of hippocampus-related brain injury in T2D (Bingham et al., 2002; Hu et al., 2019).
- *Neuroprotection from protein misfolding in cerebral hypoperfusion concurrent with Metabolic Syndrome. A translational perspective* (Luaces et al.): Metabolic syndrome (MetS) and T2D are considered risk factors for chronic cerebral hypoperfusion (CCH) and neurodegeneration (Herrera et al., 2018). Protein misfolding in CCH is a potential mechanism that can lead to either Alzheimer's disease (AD) or vascular cognitive impairment and dementia (VCID). The authors present an updated revision of preclinical findings, discussing clinical implications and proposing new experimental approaches from a translational perspective. Several animal paradigms and CCH markers have illustrated how MetS or T2D are related to CCH due to decreased cerebral blood flow, which can trigger AD or VCID via protein misfolding and aggregation.
- *Neuroprotection in metabolic syndrome by environmental enrichment. A lifespan perspective* (Kobiec et al.): MetS—concurrent obesity, hypertension, dyslipidemia, and hyperglycemia—is a global health problem with detrimental neurological consequences (Herrera et al., 2018). Early childhood is crucial in neurodevelopment, while neuroplastic changes, sensitive to environmental input, take place all over life-span. The authors present and discuss evidence showing that environmental enrichment (EE) stands as a promising non-invasive therapeutic approach. Principles of the EE, first designed for animal housing, are applied in cognitive, sensory, social, and physical stimulation programs for humans. The chapter presents milestones in neurodevelopment, along with evidence on how MetS affects neurodevelopment at each life stage and the contributions that EE models can provide to improve health over the lifespan.

Prevention is by far the best medicine, and MetS is highly preventable. Yet, this demands changing habits and lifestyle. Paraphrasing Hippocrates: “helping someone who wishes for good health is possible only if he is ready to do away with the reasons for his illness.”

## Author contributions

MO-L: Conceptualization, Investigation, Project administration, Supervision, Writing—original draft, Writing—review and editing. AM: Investigation, Project administration, Supervision, Writing—review and editing. EB: Investigation, Supervision, Writing—review and editing. FC: Investigation, Project administration, Supervision, Writing—review and editing.
